# Established and emerging treatments for diabetes-associated lower urinary tract dysfunction

**DOI:** 10.1007/s00210-022-02249-9

**Published:** 2022-05-12

**Authors:** Betül R. Erdogan, Guiming Liu, Ebru Arioglu-Inan, Martin C. Michel

**Affiliations:** 1 Department of Pharmacology, Faculty of Pharmacy, Izmir Katip Celebi University, Izmir, Turkey; 2 Department of Surgery, MetroHealth Medical Center, Case Western Reserve University, Cleveland, OH USA; 3 Department of Pharmacology, Faculty of Pharmacy, Ankara University, Ankara, Turkey; 4 Department of Pharmacology, University Medical Center, Johannes Gutenberg University, Mainz, Germany

**Keywords:** Bladder, Prostate, Diabetes, Insulin, Sodium-glucose transporter 2 inhibitors

## Abstract

Dysfunction of the lower urinary tract (LUT) including urinary bladder and urethra (and prostate in men) is one of the most frequent complications of diabetes and can manifest as overactive bladder, underactive bladder, urinary incontinence, and as aggravated symptoms of benign prostate hyperplasia. We have performed a selective literature search to review existing evidence on efficacy of classic medications for the treatment of LUT dysfunction in diabetic patients and animals, i.e., α_1_-adrenoceptor and muscarinic receptor antagonists, β_3_-adrenoceptor agonists, and phosphodiesterase type 5 inhibitors. Generally, these agents appear to have comparable efficacy in patients and/or animals with and without diabetes. We also review effects of antidiabetic medications on LUT function. Such studies have largely been performed in animal models. In the streptozotocin-induced models of type 1 diabetes, insulin can prevent and reverse alterations of morphology, function, and gene expression patterns in bladder and prostate. Typical medications for the treatment of type 2 diabetes have been studied less often, and the reported findings are not yet sufficient to derive robust conclusions. Thereafter, we review animal studies with emerging medications perhaps targeting diabetes-associated LUT dysfunction. Data with myoinositol, daidzein, and with compounds that target oxidative stress, inflammation, Rac1, nerve growth factor, angiotensin II receptor, serotonin receptor, adenosine receptor, and soluble guanylyl cyclase are not conclusive yet, but some hold promise as potential treatments. Finally, we review nonpharmacological interventions in diabetic bladder dysfunction. These approaches are relatively new and give promising results in preclinical studies. In conclusion, the insulin data in rodent models of type 1 diabetes suggest that diabetes-associated LUT function can be mostly or partially reversed. However, we propose that considerable additional experimental and clinical studies are needed to target diabetes itself or pathophysiological changes induced by chronic hyperglycemia for the treatment of diabetic uropathy.

## Introduction

Diabetes mellitus (DM) is a disease of high and further growing global prevalence (World Health Organization 2016). It leads to complications in the cardiovascular, renal, and ocular system that cause major morbidity and mortality and, thereby, constitute a major challenge to the healthcare system (Harding et al. 2019). Diabetes is also associated with an impaired function of lower urinary tract (LUT), including the bladder and urethra (and prostate in men) (Liu and Daneshgari 2014). LUT dysfunction (LUTD) often leads to LUT symptoms (LUTS) encompassing storage, voiding, or post micturition symptoms. LUTD in general and urinary bladder dysfunction are estimated to occur in 80% and 50% of diabetic patients, respectively (Daneshgari and Moore 2006; Daneshgari et al. 2009). The presence of diabetes is associated with a greater likelihood of experiencing LUTD and with a greater severity of such dysfunction. For instance, the average severity of LUTS associated with benign prostatic enlargement increases with age and they are worse in diabetic patients than in those without, i.e., a man with diabetes mathematically has worse symptoms comparable to those of a man without diabetes but 12 years older (Michel et al. 2000). Diabetes also is associated with a greater reduction of nocturia-related quality of life in men with LUTS attributed to an enlarged prostate (Michel et al. 2020).

Data from animal models of acquired diabetes support the idea that the above associations represent cause-effect relationships. Experimental diabetes can cause LUTD including overactive bladder syndrome (OAB) or, in advanced/late stages of the condition, underactive bladder syndrome (UAB) (Liu and Daneshgari 2014; Oger-Roussel et al. 2014; Tatemichi et al. 2015; Masuda et al. 2020). Similarly, numerous studies have reported an enlargement of the urinary bladder in animal models of type 1 DM (T1DM) (Arioglu Inan et al. 2018); a similar enlargement occurs in some but not other animal models of type 2 DM (T2DM) (Ellenbroek et al. 2018). A reduced size of the prostate was found in multiple studies in T1DM animals (Latifpour et al. 1991; Fukumoto et al. 1993; Wang et al. 2000; Yono et al. 2005), whereas a decrease (Atalay et al. 2017) or increase (Elabbady et al. 2016) was observed in T2DM patients, depending on the blood glucose levels.

**Fig. 1:**
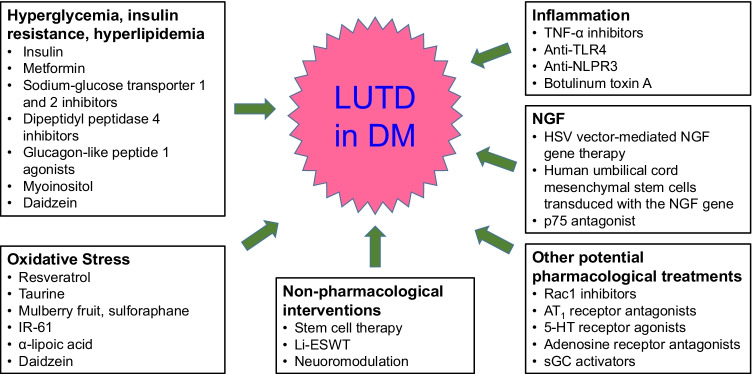
Established and emerging treatments for diabetes-associated lower urinary tract dysfunction (LUTD) in diabetes mellitus (DM)

Diabetes-associated LUTD could be managed by four, not mutually exclusive approaches: established treatments of LUT dysfunction assuming that they are similarly effective in patients with and without diabetes; antidiabetic treatments assuming that a normalization of glucose levels will also normalize LUT morphology and function; emerging treatments of LUTD by targeting pathophysiological changes in diabetes; and nonpharmacological interventions for individuals in whom pharmacological treatment is not effective. These options will be discussed hereafter based on a selective literature search and are summarized in Fig. 1.

## Effects of established medications on LUTD in diabetes

Guideline-recommended treatments of LUTD include α_1_-adrenoceptor antagonists, 5α-reductase inhibitors, and phosphodiesterase type 5 (PDE5) inhibitors (particularly in the context of benign prostatic enlargement) (Oelke et al. 2012) and muscarinic receptor antagonists and β_3_-adrenoceptor agonists (particularly in the context of OAB) (Gratzke et al. 2015); some of these have been evaluated in diabetic patients as summarized in Table 1. In most cases, clinical comparison of effects between diabetic and nondiabetic patients is based on exploratory and often post hoc analysis of studies primarily designed for other purposes. Some animal studies were designed to explore effects of medications on diabetes-associated LUTD.

**Table 1:** Key clinical studies on LUT function in diabetic patients

Drug class	Compound	Study	Main finding	Reference
α1-Adrenoceptor antagonists	Tamsulosin	Observational study	0.7-point smaller reduction in IPSS in diabetic patients	Michel et al. 2000
	Alfuzosin, doxazosin, tamsulosin, terazosin	Retrospective analysis	IPSS reduction by 7.5 and 3.1 in diabetic and nondiabetic patients respectively	Bozlu et al. 2014
5α-reductase inhibitors	Dutasteride and finasteride	Population-based database analysis	Increased risk to develop new onset T2DM	Wei et al. 2019
Muscarinic receptor antagonists	Darifenacin	Observational study	Smaller improvement of urinary dysfunction in diabetic patients	Schneider et al. 2013
	α-Blocker + tolterodine	Observational study	Comparable IPSS improvement in diabetic and nondiabetic patients	Höfner et al. 2010
	α-Blocker + solifenacin	Case series	Smaller OAB symptom improvement in diabetic patients	Obata et al. 2013
	Solifenacin	Prospective open-label study	Comparable effect on diabetic OAB and idiopathic OAB	Choi et al. 2018
	Darifenacin or solifenacin vs. oxybutynin	Retrospective cohort study	Increased risk for developing diabetes in patients taking darifenacin or solifenacin	Selig et al. 2020

### α_1_-Adrenoceptor antagonists

Clinical and experimental studies have tested α_1_-adrenoceptor antagonists in diabetic patients and animals. A large noninterventional study in men with LUTS compared beneficial effects of tamsulosin in 1290 men with and 8566 without diabetes (Michel et al. 2000). While tamsulosin reduced symptom severity assessed as International Prostate Symptom Score (IPSS) by 10.5 points in the overall group, a diagnosis of diabetes was associated with a 0.7 points smaller reduction in a multivariate analysis. This may be of limited clinical relevance because a symptom change of at least 3 points is assumed to be noticeable by a patient (Barry et al. 1995). A retrospective analysis of men with LUTS treated with alfuzosin, doxazosin, tamsulosin, or terazosin with (*n* = 60) and without diabetes (*n* = 221) reported reductions in IPSS by 7.5 and 3.1 points, respectively (Bozlu et al. 2004); however, these data are not easily interpretable because diabetics had a greater IPSS at baseline, no baseline adjustments were made, and reported statistical analysis only compared baseline with post-treatment data within each group. Taken together, these data indicate that α_1_-adrenoceptor antagonists have comparable efficacy in alleviating LUTS to a clinically relevant extent in men with as compared to those without diabetes.

Studies in diabetic animals primarily explored whether α_1_-adrenoceptor antagonists can improve dysfunction of the bladder and urethra. Most used the streptozotocin (STZ) model of T1DM, but one used Zucker diabetic fatty rats as a model of T2DM (Tatemichi et al. 2015). An increased bladder capacity is a frequent feature of animal models of diabetes (Ellenbroek et al. 2018), apparently reflecting the need to handle increased volumes of urine related to diabetic polyuria. While an increased bladder capacity clinically is more typical for UAB than OAB, it remains unclear whether this alone constitutes proof of an UAB. In STZ-injected rats, chronic administration of silodosin attenuated the diabetes-associated increases in bladder capacity and residual urine (Yonekubo et al. 2017). Acute administration of tamsulosin in the rat STZ model did not affect bladder capacity or residual urine (Sekido et al. 2020), and acute administration of silodosin did not alter bladder capacity or residual volume in Zucker diabetic fatty rats (Tatemichi et al. 2015). Acute administration of terazosin (Torimoto et al. 2005) or tamsulosin (Chen et al. 2014) was reported to improve urethral function in STZ-injected rats, i.e., reduced the elevated urethral perfusion pressure.

### 5α-Reductase inhibitors

5α-Reductase inhibitors are frequntly used in the treatment of LUTS suggestive of benign prostatic hyperplasia (BPH). Little clinical evidence is available on 5α-reductase inhibitor effects in DM. In population-based cohort studies from the UK and Taiwan, use of a 5α-reductase inhibitors was associated with a greater risk to develop new-onset T2DM as compared to use of tamsulosin; the hazard ratios for dutasteride and finasteride relative to tamsulosin were 1.32 [95% confidence interval 1.08; 1.61] and 1.26 [1.10; 1.45], respectively, in the UK with similar numbers in Taiwan (Wei et al. 2019). The mechanistic plausibility of these findings is supported by two animal studies: 5α-reductase knock-out mice and Zucker diabetic fatty rats treated with finasteride reported that such inhibition predisposed to insulin resistance (Livingstone et al. 2015). While we did not identify studies comparing the efficacy of 5α-reductase inhibitors in men with and without diabetes, these data suggest that if anything their use should make the diabetes-associated part of male LUT symptoms worse.

### Phosphodiesterase type 5 inhibitors

PDE5 inhibitors are primarily indicated for the treatment of erectile dysfunction. While various PDE5 inhibitors have been tested in phase 1 and 2 studies in LUTS patients, only tadalafil was approved for the treatment of male LUTS attributed to benign prostatic enlargement. While we are not aware of clinical studies comparing its efficacy in men with LUTS with and without concomitant diabetes, a pooled analysis of 11 randomized clinical trials found that its efficacy in the treatment of erectile dysfunction is slightly lower in those with than those without diabetes (34.7% vs. 36.9% attaining normal erectile function) (Lewis et al. 2005), possibly reflecting diabetic neuropathy. In a clinical study in men with LUTS and OAB, diabetes was present as a comorbidity in 10 of 44 responders but in 0 of 9 nonresponders (Matsuo et al. 2020), indicating that the effects of tadalafil and possible other PDE5 inhibitors in LUTS may not be attenuated by concomitant diabetes. Seven weeks after STZ injection in rats, a 7-day treatment with tadalafil restored prolonged inter-contraction intervals and diminished bladder blood flow to values comparable to those in control animals (Gotoh et al. 2018). In a follow-up study, tadalafil restored urethral relaxation and detrusor contraction (Gotoh et al. 2019). Restoration of detrusor function by tadalafil in UAB observed at late time points after administration of STZ was also reported by others (Masuda et al. 2020). These data indicate beneficial effects of tadalafil in diabetic uropathy. Based on the proposed role of diminished perfusion in bladder dysfunction (Michel et al. 2015a; Thurmond et al. 2016), they also provide evidence that the beneficial effects may at least partly be due to improved perfusion.

### Muscarinic receptor antagonists

The effect of muscarinic receptor antagonists in patients with OAB has been compared between those with and without concomitant diabetes, largely in noninterventional studies. In male and female OAB patients receiving darifenacin (532 with 1315 without concomitant diabetes), the presence of diabetes was associated with smaller improvements of urgency, incontinence, frequency, and nocturia in a multivariate analysis adjusting for gender, age, and baseline symptoms, but the impact of diabetes was small relative to the overall symptom improvement by darifenacin (Schneider et al. 2013). Two other noninterventional studies have compared the effects of adding a muscarinic receptor antagonist in men with and without diabetes and with persisting storage symptoms despite treatment with an α-blocker. In a cohort of 741 men, addition of tolterodine caused comparable additional improvements in IPSS in men with and without diabetes (Höfner et al. 2010). In a similar study, addition of solifenacin was associated with a smaller improvement of the OAB Symptom Score in a multivariate analysis in the presence of diabetes of a total group of 130 men (0.6 vs. 2.1 points of the OAB Symptom Score) (Obata et al. 2013). One prospective open-label study compared effects of solifenacin in 81 women with diabetes-associated OAB with those in 160 women with idiopathic OAB and found a comparable degree of symptom improvement in both groups (Choi et al. 2018). While small sample sizes make interpretation of the data difficult, it appears that muscarinic antagonists may be slightly less effective in those with OAB symptoms with as compared to those without concomitant diabetes. However, group differences appear to be small, and the efficacy of the muscarinic antagonists in the diabetic groups remained of clinical relevance. Animal studies support this conclusion: Goto-Kakizaki rats, a T2DM model, exhibited detrusor overactivity that was markedly reduced by solifenacin (Oger-Roussel et al. 2014). An interesting alternative explanation for slightly smaller efficacy of the OAB medications in those with diabetes relates not to pharmacodynamic differences but rather to a reported lesser adherence to muscarinic antagonists prescribed as OAB medication in obese patients (Lua et al. 2017), a feature common in T2DM. On the other hand, analysis of a large database has found that treatment with a muscarinic antagonist moderately selective for M_3_ receptors such as darifenacin or solifenacin as compared to oxybutynin increased the risk for developing diabetes with a hazard ratio of 1.57 [1.48; 1.67] and 1.29 [1.24; 1.35], respectively (Selig et al. 2020); these data do not allow conclusions whether darifenacin or solifenacin promote diabetes or protect less efficiently than oxybutynin; the impact on diabetes-associated LUT dysfunction remains to be assessed.

### β_3_-Adrenoceptor agonists

While we are not aware of clinical data with a β_3_-adrenoceptor agonist in OAB patients with and without diabetes, chronic ischemia/hypoxia is proposed to play a pathophysiological role in bladder dysfunction (Michel et al. 2015a; Thurmond et al. 2016). Against this background, a study reported that treatment with the experimental β_3_-adrenoceptor agonist CL 316,243 attenuated vascular damage from ischemia/reperfusion injury in mice and in isolated human endothelial cells (Bubb et al. 2021). Although brown adipose tissue is present in humans to a limited extent only, administration of the β_3_-adrenoceptor agonist mirabegron enhanced thermogenesis in humans (Cypess et al. 2015), an effect that if anything should improve glycemic control. More recent work from the same group found that mirabegron also improved insulin sensitivity in healthy volunteers (O'Mara et al. 2020). Whether such effects also relate to bladder dysfunction remains to be established.

## Effects of antidiabetic treatments on LUTD in diabetes

Not all complications of diabetes are equally well prevented by glucose lowering (Beckman and Creager 2016). This raises the question whether antidiabetic medications substantially improve LUT function. Conceptually, this could be a reversal of dysfunction in established diabetes or a prevention of dysfunction in new onset diabetes (operationally defined as starting within one week after start of diabetes); the latter is not a realistic clinical setting with the possible exception of T1DM. We are unaware of clinical studies exploring the effect of antidiabetic medication on LUT function in a treatment or prevention setting, but various animal studies have been reported in both settings. A key feature of LUT manifestations of diabetes in experimental animals is an enlargement of the urinary bladder; this is consistently found in all animal models of T1DM, and in some but not others of T2DM (Arioglu Inan et al. 2018; Ellenbroek et al. 2018). Therefore, many experimental studies have reported bladder weight as outcome parameter for antidiabetic treatment effects on LUT function. Most studies have applied insulin in the T1DM model of STZ-induced diabetes (mostly in rats), whereas information on treatments typically used in the management of T2DM is emerging only recently.

### Insulin

Only limited clinical data are available for the effect of insulin on bladder dysfunction in diabetic individuals. The Diabetes Control and Complications Trial and Epidemiology of Diabetes Interventions and Complications (DCCT/EDIC) study participants were evaluated with follow-up studies for the development of urinary system complications. In a 10-year follow-up study, LUTS were evaluated in 591 men with a T1DM history for ~ 20 years. One hundred fifteen men (mean 44.6 ± 6.6 years of age) reported to have moderate/severe LUTS. The severity and prevalence of LUTS markedly increased after age of 50 s; however, blood glucose control with either intensive (3 or more times per day or continuous injection with insulin pump) or conventional (1 or 2 injection per day) insulin treatment did not affect the severity of LUTS of the cohort (Van Den Eeden et al. 2009). However, considering the greater prevalence of LUTS in older ages (Irwin et al. 2006), it is not plausible to extrapolate this finding to the general population. In a 10- and 17-year follow-up study, urinary incontinence (at least once a week) in 471 women with T1DM was evaluated as an incident reported at 17-year but not at 10-year follow-up questionnaire. Sixty-four women developed urinary incontinence in a 7-year time period. Mean HbA1c levels up to EDIC year 10 were higher (8.4 ± 1.2% vs 7.9 ± 1.1%) in women who developed urinary incontinence compared with the women who did not develop urinary incontinence. Lower HbA1c levels were associated with a reduced odds ratio of urinary incontinence incident (Lenherr et al. 2016) whereas an earlier study revealed no association between HbA1c levels and incontinence in women with both T1DM and T2DM (Lee et al. 2013). Thus, these clinical data allow only limited conclusions on the effect of treatment with insulin on LUTS in diabetic patients.

Considerably more information is available from the STZ model of T1DM in rats. The high glucose levels following injection of STZ typically lead to polyuria caused by osmotic diuresis. Treatment with insulin consistently reduced diabetic polyuria in a prevention (Longhurst et al. 1991; Daneshgari et al. 2006; Melman et al. 2009; Gotoh et al. 2021) and in a treatment setting (Longhurst et al. 1991; Xiao et al. 2015). This is relevant because some investigators proposed that polyuria is the main cause of bladder enlargement in experimental diabetes, although some analyses did not confirm that (Ellenbroek et al. 2018; Yesilyurt et al. 2019). The effect of insulin treatment on bladder weight has mostly been studied in a prevention setting, i.e., starting within 1 week after induction diabetes. With one exception (Evcim et al. 2015), these studies consistently reported that early administration of insulin in a dose to avoid major elevations in blood glucose prevented or at least strongly attenuated diabetes-associated bladder enlargement (Longhurst et al. 1991; Yono et al. 2005; Christ et al. 2006; Daneshgari et al. 2006; Melman et al. 2009). Similarly, a more limited number of studies in a treatment setting reported that insulin administration starting 3 (Xiao et al. 2015) or 8 weeks after STZ injection (Longhurst et al. 1991; Fukumoto et al. 1994), i.e., at a time when bladder enlargement had fully developed (Arioglu Inan et al. 2018), largely reversed bladder enlargement. Interestingly, these reversal data indicate that bladder denervation, possibly occurring as part of diabetic polyneuropathy, may not be a main cause of bladder enlargement as nerve regeneration is not expected within the time frames in which insulin could reverse bladder enlargement.

The in vivo analysis of bladder function in diabetes has been based on experiments using metabolic cages (Longhurst et al. 1991; Daneshgari et al. 2006; Xiao et al. 2015; Gotoh et al. 2021) and/or cystometry (Christ et al. 2006; Daneshgari et al. 2006; Melman et al. 2009; Xiao et al. 2015; Gotoh et al. 2021). In a treatment (Xiao et al. 2015) and in a prevention design (all other studies), it was observed that insulin reduced number of micturitions and mean voided volume per micturition, which is to be expected if insulin lowers glucose levels and thereby reduces diabetic polyuria. Various cystometric parameters including bladder capacity were also improved. Insulin also prevented the diabetes-associated increases in residual urine (Christ et al. 2006; Daneshgari et al. 2006; Wang et al. 2010). The effects of treatment with insulin on in vitro parameters of bladder function have been studied less often and yielded conflicting results: contractile responses to a muscarinic agonist were increased in diabetes, but this was attenuated in strips from insulin-treated animals in one (Longhurst et al. 1991) but not another study (Evcim et al. 2015). Of note, the Longhurst study consistently observed an improvement with insulin administered both in a prevention and a treatment setting and reported similar findings if ATP was used as the contractile agonist. One group reported that STZ alone increased expression of muscarinic receptors in the bladder and that insulin starting early after STZ administration prevented that (Latifpour et al. 1992). In a follow-up study, they reported that insulin administration starting 8 weeks after STZ also attenuated the increased expression of muscarinic receptors (Fukumoto et al. 1994). In a similarly designed study, they also reported that insulin reversed the STZ-induced upregulation of endothelin receptors in the bladder (Saito et al. 2000b). Another study found that diabetes lasting for 6 or 10 weeks led to altered length/tension curves (Starling mechanism) and that was prevented by treatment with insulin (Wang et al. 2010). Finally, it was found that treatment with insulin can also prevent diabetes-associated dysfunction of the urethra (Gotoh et al. 2021).

Over a period of two decades, a group from New Haven, CT, has continuously explored the effects of treatment with insulin on STZ effects in the rat prostate and, to a lesser extent vas deferens. In their initial experiments, they started insulin treatment 3 days after injection of STZ. This caused the expected increase in urine output; however, in contrast to findings in the bladder (Arioglu Inan et al. 2018), this did not enlarge but rather reduce prostate weight to an extent at least as much as the reduction in body weight, the latter being a typical feature of STZ administration. This was accompanied by a major reduction in the density of muscarinic receptors (Latifpour et al. 1991) and of β-adrenoceptors (Gousse et al. 1991) as detected in radioligand binding studies. Treatment with insulin prevented all of these alterations. Of note, the radioligand used for detection of β-adrenoceptors, [^3^H]-dihydroalprenolol, was used in concentrations that allow detection of β_1_- and β_2_-, but not β_3_-adrenoceptors (Niclauß et al. 2006). Apparently, the detected receptors were an almost homogenous population of β_2_-adrenoceptors in all three groups. The muscarinic receptors being detected apparently largely belonged to the M_3_ subtype in all three groups, whereas several other groups reported a presence of mainly M_1_ receptors in the prostate (Witte et al. 2008). A follow-up study used the same model but started treatment with insulin 8 weeks after induction of diabetes. When measured 16 weeks after administration of STZ (8 weeks after start of insulin treatment), prostate weight was also reduced, which was accompanied by reduced serum testosterone levels (Fukumoto et al. 1993). Treatment with insulin reversed these changes and the reduction of muscarinic and β-adrenergic binding sites. Applying the same study design, the weight of the vas deferens was also reduced whereas the density of muscarinic binding sites in this tissue was increased in insulin-treated animals (Kamai et al. 1994). The affinity estimates for multiple muscarinic antagonists in prostate and vas deferens were correlated only poorly, indicating that the difference between the two tissues may reflect a different dominant subtype of muscarinic receptors. In an additional study of similar design, treatment with insulin also reversed the upregulation of endothelin-1 binding sites in the vas deferens, apparently reflecting a homogenous population ET_A_ receptors (Saito et al. 1996). Subsequent studies also using a treatment design reported that treatment with insulin reversed greater expression of fibroblast growth factor-2 (also known as basic fibroblast growth factor) (Wang et al. 2000), greater expression of transforming growth factor-β (TGF-β), and the related mRNAs, TGF-β1 and TGF-β2 (Ikeda et al. 2000), and increased expression of endothelin receptors (about 80% ET_A_ and 20% ET_B_ receptors in all groups) (Saito et al. 2000a). BB/Wor rats spontaneously develop T1DM. Studies in 8, 16, and 32-week BB/Wor rats as well as those in 32-week-old BB/Wor rats additionally injected with STZ found that diabetes increased the size of the urinary bladder, the vas deferens, the ureter, and, to a lesser extent, the kidney whereas it reduced the size of the ventral prostate and did not change sizes of the dorsolateral prostate, seminal vesicles, testes, or adrenal glands; all diabetes-associated changes were attenuated to some degree by insulin administration dosed to maintain euglycemic levels (Yono et al. 2005). Extending the work in BB/Wor- and STZ-injected BB/Wor rats, the same group also reported on microarray analysis (Yono et al. 2008). The hyperglycemic state was associated with altered expression levels of 856 genes, of which 35 were related to cell growth, proliferation, and cell death in the ventral prostate; such alterations were largely attenuated upon treatment with insulin.

Taken together, these data support the idea that in the STZ-induced model of T1DM, treatment with insulin can largely prevent and reverse diabetes-associated LUT dysfunction, organ sizes, and expression levels of various genes. The importance of these findings is that they establish that the diabetes-associated alterations are largely reversible within certain period of diabetes duration. Moreover, they highlight that tissues within the LUT, most importantly bladder and prostate, exhibit differential regulation by diabetes. A key limitation of these findings is that they are based on an animal model of T1DM, mostly STZ-injected rats. Alterations of bladder size in animal models of T2DM apparently are limited to some models, and the occurrence of bladder enlargement in these models apparently is not related to the degree of hyperglycemia (Ellenbroek et al. 2018). This raises the question whether such changes also be prevented or reversed by treatments other than insulin; such data have only emerged recently.

### Metformin

Metformin is the cornerstone of medical treatment of T2DM. It has been studied in some animal models of LUT dysfunction, but interestingly not those of diabetes. In a rat model of testosterone-induced prostate enlargement, treatment with metformin at least partly prevented increases in prostate size (Mosli et al. 2015). It also prevented testosterone-induced increases in estrogen receptor-α and decreases in estrogen receptor-β expression, while not affecting expression of the androgen receptor or of 5α-reductase. Other investigators used rats with partial bladder outlet obstruction and started metformin treatment within 3 days after induction (Chen et al. 2021). In the early phase (2 weeks after induction), metformin lowered baseline bladder pressure and reduced inflammatory reactions. In the chronic phase (9 weeks after induction), metformin improved the reduced bladder compliance, ameliorated fibrosis, and inhibited autophagy. In contrast to these two studies using a prevention design, other investigators induced bladder dysfunction in mice by administering methylglyoxal, a reactive carbonyl species present in high concentrations in the blood of diabetic patients; metformin was administered in the final 2 weeks of a 12-week exposure to methylglyoxal, i.e., in a treatment design (Oliveira et al. 2021). This model is not associated with changes in body weight, water consumption, or blood glucose levels, either in the absence or presence of metformin. However, it is associated with a reduced bladder weight that was not affected by metformin. Nonetheless, metformin reversed elevations in serum methylglyoxal and advanced glycation end products. It also reversed increases in urothelial thickness and collagen content (detrusor thickness was not altered in this model), in total voided volume, volume per void, basal pressure, nonvoiding contractions, bladder capacity, and residual urine leading to an increased voiding efficiency. It reversed increased contractile responses to αβ-methylene-ATP and electrical field stimulation but did not affect reduced contractile responses to carbachol. Taken together, these three studies demonstrate that metformin can have beneficial effects in euglycemic animal models of LUT dysfunction; however, they do not allow conclusions on possible effects in animal models of diabetes.

### Sodium-glucose transporter 2 inhibitors

Sodium-glucose transporter 2 (SGLT2) inhibitors such as empagliflozin (Michel et al. 2015b) are an innovative class of oral antidiabetic agents that not only lower glucose levels but also have nephroprotective effects (Wanner et al. 2018) and reduce mortality in diabetic and nondiabetic heart failure patients with a reduced ejection fraction (McDonagh et al. 2022). We have tested the SGLT2 inhibitor dapagliflozin in a randomized, blinded study in a rat model of T2DM that is based on a combination of low-dose STZ injection with a high fat diet (HFD) (Yesilyurt et al. 2019). The study exposed 5-week-old rats to a HFD and an injection of STZ after 4–5 weeks on that diet; a minimum of 6 weeks after the STZ injection, treatment with dapagliflozin started and was maintained for another 12–15 weeks. Interpretation of this study is hampered by the fact that mean glucose levels peaked around 300 mg/dl but declined to 205 mg/dl in the diabetic group at study end. The effectiveness of dapagliflozin was confirmed by an increase in diuresis. Under these conditions of only mild hyperglycemia, the investigators did not observe major changes in bladder weight, or contractile responses to carbachol or KCl, or relaxant responses to several general or β_2_- or β_3_-adrenoceptor-selective agonists or forskolin. While not approved for the treatment of T1DM, SGLT2 inhibitors have shown beneficial effects in animal models of T1DM (Michel et al. 2015b). Therefore, effects of empagliflozin were studied in STZ-injected rats, in a preventive setting in female rats (Yesilyurt et al. 2021), and a treatment setting in male rats (Michel et al. 2021); the latter study also tested the dipeptidyl-peptidase 4 inhibitor linagliptin, a drug not expected to affect glucose homeostasis in this model. In the preventive study, empagliflozin lowered glucose levels, albeit not to normoglycemic levels, and prevented increases in urine output and bladder weight. Contractile responses to carbachol or KCl measured in isolated bladder strips or relaxant responses to general or β_2_- or β_3_-adrenoceptor-selective agonists or forskolin were not substantially affected by diabetes or empagliflozin. While the treatment study found a similar decrease of blood glucose by empagliflozin (not by linagliptin) as in the prevention study, STZ-associated increases in bladder weight were not affected by either drug. Whether the differences between the two studies reflects those in prevention vs. treatment setting, those between female and male rats or unknown other causes remains unclear. The effect of SGLT2 inhibitors on human LUT function remains to be established.

### Dipeptidyl-peptidase 4 inhibitors and glucagon-like peptide 1 receptor agonists

Chronic hypoperfusion due to atherosclerosis of pelvic arteries is considered as a cause of bladder dysfunction (Michel et al. 2015a; Thurmond et al. 2016). One study explored effects of a 4-week treatment with the dipeptidyl-peptidase 4 inhibitor anagliptin and the glucagon-like peptide 1 (GLP-1) receptor agonist liraglutide in a preventive setting in a rat model of acute bilateral internal iliac artery ligation (Hotta et al. 2020). Neither the ligation nor the treatments affected glucose levels or bladder weight. The ligated animals exhibited the expected reductions in bladder blood flow, which were improved with anagliptin. Inter-contraction intervals in cystometric studies were longer in ligated and ligated animals treated with liraglutide, but ligated rats treated with anagliptin exhibited inter-contraction intervals as sham-operated animals. These data indicate that dipeptidyl-peptidase 4 inhibitors may have beneficial effects on bladder function in normoglycemic animals. Other investigators tested the GLP-1 analogs exendin-4 and liraglutide in an acute mouse model of neurogenic voiding dysfunction induced by middle cerebral artery occlusion, in this case in diabetic db/db mice (Li et al. 2016). While this study did not assess glucose levels after ligation and/or any of the treatments, db/db mice are an established a model of T2DM (Kong et al. 2013). The ligation led to a reduced number of voids per 10-min period and an increase in number of nonvoiding contractions of the bladder; when applied in a prevention design, exendin-4 and liraglutide attenuated both effects of ligation. Although the animals being studied apparently were diabetic, this model probably reflects neurogenic dysfunction in diabetic animals and not diabetes-associated LUT dysfunction. Effects of dipeptidyl-peptidase 4 inhibitors and GLP-1 analogs in diabetes-associated bladder dysfunction remain to be studied.

## Emerging pathophysiology-based treatments of LUTD in diabetes

Other than general LUTD or antidiabetic drugs, a third possible approach would be medications specifically addressing diabetes-associated pathophysiology in LUTD. The theoretical feasibility of this approach is demonstrated by drugs specifically targeting diabetic retinopathy (Beckman and Creager 2016). Candidates for the treatment of diabetic uropathy are discussed hereunder.

### Oxidative stress

Oxidative stress may be one of the underlying mechanisms in diabetic bladder dysfunction as well as other complications of the diabetes (Wang et al. 2016). Multiple studies have reported that oxidative stress increases in bladder tissues in diabetes (Changolkar et al. 2005; Kanika et al. 2011; Ha et al. 2013; Chen et al. 2015; Elrashidy and Liu 2019; Lin et al. 2020; Wang et al. 2021; Laddha and Kulkarni 2022). Oxidative stress has also been reported from clinical studies of various types of LUTD other than diabetes (Antunes-Lopes et al. 2019; Matsuo et al. 2020), and the urinary presence of oxidative stress indicators such as 8-hydroxy-2′-doexyguanosine has been proposed as biomarker for LUTS in general (Kageyama et al. 2018; Matsumoto et al. 2019). Interestingly, treatment for 14 days with the antioxidants resveratrol (10 mg/kg/day, p.o.) and taurine (1 g/kg/day, p.o.) moderately reduced blood glucose levels but almost fully prevented diabetes-associated bladder enlargement at early stages of diabetes in male rats with T2DM (Tsounapi et al. 2019). We found increased nitrotyrosine levels in the bladders of 9-week diabetic mice (Elrashidy et al. 2019), 20-week (Xiao et al. 2013), and 44-week (Elrashidy and Liu 2019) diabetic rats. Increased lipid peroxidation products and aldose reductase were found to be associated with the decrease in detrusor muscle contractility in alloxan-induced diabetic rabbits (Changolkar et al. 2005). Increased oxidative stress can damage smooth muscle cells (Kanika et al. 2011), interrupt neurotrophins necessary for neuron survival (Whitmire et al. 2011), and induce apoptosis (Ha et al. 2013; Chen et al. 2015; Lin et al. 2020), which could contribute to diabetic cystopathy. Several antioxidant agents were studied on diabetic bladder dysfunction. The cyanidin-3-O-β-_D_-glucopyranoside fraction of mulberry fruit treatment in STZ-diabetic male rats has markedly ameliorated bladder capacity, maximal detrusor pressure, contraction frequency, and contaction interval through reducing oxidative stress and apoptosis (Ha et al. 2013). Similarly, grape seed proanthocyanidin extract as a preventive treatment was found to decrease oxidative stress markers and apoptosis in STZ-induced diabetic female rat bladder; a beneficial effect of the extract in mitigating functional and histological changes of bladder was attributed to activation of antioxidant nuclear erythroid related factor2 (Nrf2) mediated pathway (Chen et al. 2015). Supporting the role of Nrf2 in pathogenesis of diabetic bladder, prophylactic antioxidant treatment with the Nrf2 activator sulforaphane remarkably reduced bladder capacity and mictirituon duration by reducing reactive oxygen species (ROS) levels and related endoplasmic reticulum stress and apoptosis (Lin et al. 2020). Increased intracellular and mitochondrial ROS levels were accompanied by increased apoptosis of bladder smooth muscle cells in 11-week STZ-induced diabetic female rats. IR-61, is a fluorescent dye with antioxidant effect, treatment (1.6 mg/kg/week, i.p.) for 10-week markedly reduced oxidative stress and apoptosis in bladder tissue and improved the bladder dyfunction compared to diabetic rats (Wang et al. 2021). Considering the role of oxidative stress in pathogenesis of diabetic bladder and beneficial effects of antioxidant agents, it is reasonable to target oxidative stress to treat bladder complications in diabetes.

### Inflammation

Inflammation may play a role in the pathophysiology of diabetic bladder dysfunction as in other complication of diabetes (Navarro and Mora 2005). The role of several inflammatory mediators in diabetic bladder dysfunction has been studied. Elevated tumor necrosis factor-α (TNF-α) levels in serum and bladder tissue were shown in a genetic T2DM model, DKO female mice. TNF-α was shown to be implicated in bladder dysfunction exhibiting as overactivity in early (12-week) stage and hypoactivity in late (20-week) stages of diabetes. Six-week-old animals were administered a TNFRI (inhibitor of TNF-α) (2 mg/kg, twice per week, i.p.) for 6 weeks, and frequency of nonvoiding contractions, voided volume, and micturition volume were improved in treated mice compared to diabetic group. Moreover, concomitant administration of TNFRI and metformin yielded a better outcome in reducing frequency of nonvoiding contractions compared to TNFRI alone (Wang et al. 2012). TLR4, an innate immune receptor, was upregulated 4 weeks after STZ injection in male mice. Elevated inflammatory and oxidative stress response due to increased TLR4 activation was found to mediate bladder hypertrophy and increased bladder contractility in diabetes. These changes were attenuated in TLR4 knockout diabetic mice compared to wild-type diabetic group (Szasz et al. 2016). The NLRP3 inflammasome was found to be activated in T1DM Akita female mice. NLRP3-mediated inflammation was implicated in decreased sense of bladder, void volume, and voiding efficiency as well as increased increased frequency of voiding and postvoid residual volume in diabetic mice compared to control. Depletion of NLPR3 gene prevented the unfavorable effect of diabetes on bladder (Hughes et al. 2019). Botulinum toxin A was approved by FDA and NICE in OAB patients who do not respond or tolerate anticholinergic drugs (Ibrahim et al. 2022). Intravesical injection of botulinum toxin A inhibits both hypersensivity of detrusor muscle and chronic inflammation. Thereafter, it has been used successfully in the treatment of diabetes-related OAB in patients, yet more studies are needed to ensure efficacy and safety of botulinum toxin A (Wang et al. 2020a). Considering the possible role of inflammation in diabetic bladder dysfunction, targeting inflammatory pathways might be beneficial to treat complications.

### Rac1

The GTPase Rac1 has a role in the oxidative stress response and also is essential for the contraction of the smooth muscle (Rahman et al. 2014). An augmented Rac1 expression and an increased retinal ROS production were found to be involved in the development of diabetic retinopathy (Mohammad et al. 2019). The expression of Rac1 was found to be increased in overall bladder of STZ-induced diabetes in rats (Laddha and Kulkarni 2022), in bladder smooth muscle, but not urothelium in mice with STZ-induced diabetes (Poladia and Bauer 2004). Another study using STZ-treated rats found that Rac1 immunoreactivity was increased in the epithelium, lamina, propria and tunica muscularis of the bladder; this was attenuated but not eliminated by insulin treatment (Evcim et al. 2015). In isolated bladder strips obtained 8 or 12 weeks after the STZ injection, the Rac1 inhibitor NSC23766 (0.1, 1, and 10 µM) attenuated carbachol-induced contraction in all groups, but the extent of inhibition was greater in the diabetic and insulin-treated than the nondiabetic rats (Evcim et al. 2015). While these data leave it open whether Rac1 is solely increased in smooth muscle or also in mucosal cells, they indicate that the role of Rac1 may specifically increase in diabetes and make Rac1 a potential target for diabetic uropathy treatment. Accordingly, inhibitors of Rac1 such as EHT1864 or NSC23766 can attenuate contraction elicited via multiple receptors in the human prostate (Wang et al. 2015) and bladder (Li et al. 2020). Moreover, inhibition of Rac1 by small molecules or by gene silencing reduced growth and actin organization of human bladder smooth muscle cells (Wang et al. 2020b). A limitation of these findings is that NSC23766 is not only a direct inhibitor of Rac1 but also a competitive antagonist of muscarinic receptors (Levay et al. 2013). This complication may be absent with EHT1864 (Li et al. 2020). Another complication is that both NSC23766 and EHT1864 showed considerable effects in Rac1 knockout cells, implying that they have Rac1-independent effects (Wang et al. 2020b). Nonetheless, the overall data suggest that Rac1 may play a role both in contraction and hypertrophy development in the LUT. Treatment with daidzein dose-dependently attenuated the increased Rac1 expression in diabetic rats (Laddha and Kulkarni 2022).

### Nerve growth factor

Nerve growth factor (NGF) and its receptors including p75 are expressed in urinary bladder and have an essential role in its innervation and maintenance of function (Ochodnicky et al. 2011; Elrashidy and Liu 2019). While several studies have explored the expression of NGF and p75 in the bladder of STZ-injected rats, the reported findings are inconclusive: several studies reported a reduced expression (Sasaki et al. 2002; Tong and Cheng 2005; Jiang et al. 2008; Nirmal et al. 2014; Elrashidy and Liu 2019), but some also reported at least transient increases (Koo et al. 1993; Steinbacher and Nadelhaft 1998). If a potential upregulation is transient as proposed by some (Koo et al. 1993), studies with few time points may have missed that. This also makes it difficult to interpret single timepoint data from other models such as fructose-fed rats (Cheng and Tong 2008). Nonetheless, these findings motivated several investigators to explore effects of interventions on bladder function and the NGF system or those directly targeting NGF. Treatment with either insulin or the sodium-glucose transporter 1 and 2 inhibitor phlorizin prevented the STZ-induced reduced expression of NGF and its receptor, p75 (Tong and Cheng 2005). Treatment with α-lipoic acid starting 6 weeks after STZ injection normalized the lowered NGF expression and bladder function (Jiang et al. 2008). After a feasibility study had demonstrated successful NGF expression in mice using a Herpes simplex virus (Goins et al. 2001), the same group reported that injection of this vector into the bladder wall 8 weeks after STZ injection restored lower NGF levels and the impaired bladder function as assessed by cystometry (Sasaki et al. 2004). Another approach to increase NGF levels has been the use of human umbilical cord mesenchymal stem cells transduced with the NGF gene in STZ diabetic rats (WenBo et al. 2017). When administered 3 days after the STZ injection, the impaired voiding function was improved. Unlike NGF, proNGF (a precursor of NGF) induces inflammation and degenerative pathways although both use the p75 receptor. In a mouse STZ model, an proNGF antibody or a small molecule p75 inhibitor were tested (Mossa et al. 2020). The p75 antagonist reduced STZ-induced bladder enlargement, whereas the proNGF antibody did not. On the other hand, the antibody normalized impaired bladder function, whereas the antagonist did not. The authors speculated that it may be less NGF levels but rather the proNGF/NGF ratio that is important for amelioration of diabetes-associated bladder dysfunction.

### Angiotensin II type 1 receptors

Angiotensin II plays a role in the pathophysiology of many diseases, and inhibitors of the angiotensin II type 1 (AT_1_) receptors are effective in various human diseases and in animal models of even more conditions (Michel et al. 2016). Specifically, AT_1_ antagonists exert antihypertrophic effects on various tissues, particularly related to smooth muscle hypertrophy. The best studied model of bladder hypertrophy is surgically induced bladder outlet obstruction, most often applied to rats. The AT_1_ antagonist telmisartan was reported to attenuate, but not abolish bladder enlargement in this model, whereas the obstruction-associated decrease in AT_1_ receptor binding was abolished (Yamada et al. 2009). Another study did not confirm the attenuation of bladder hypertrophy by telmisartan but found that telmisartan abolished the obstruction-induced increase in AT_1_ receptors and NGF in both detrusor and urothelium (Cho et al. 2012). In cystometric investigations, telmisartan attenuated the shortening of the inter-contraction intervals and abolished the increase in nonvoiding bladder contractions. Interestingly, the combined presence of bladder outlet obstruction and STZ-induced diabetes caused greater bladder enlargement and disturbance of contractile function than either condition alone in one study (Longhurst et al. 2004), but this was not confirmed in a later study by other investigators (Öztürk et al. 2002). Based on these findings, the AT_1_ antagonist valsartan (starting 2 weeks after STZ) was tested in the rats exposed to a combination of low-dose STZ and a HFD, a model of type 2 diabetes (Arioglu Inan et al. 2022). However, valsartan did not reverse bladder enlargement, nor did it affect contractile or relaxant response in isolated bladder strips. Thus, the limited data on AT_1_ antagonist are inconsistent within the obstruction model and in comparison, with diabetes.

### Serotonin receptors

Autonomic neuropathy in diabetes leads to reduced sense of bladder filling and is associated with diabetes-related bladder dysfunction (Duby et al. 2004). Central serotonin (5-HT) receptors are localized in nerve terminals and have variable effects (mostly inhibitory) in the control of micturition depending on receptor subtype and species (Ramage 2006). STZ-induced T1DM for 8 weeks in female rats resulted in reduced voiding function accompanied with increased bladder capacity and residual volume of urine and impaired external urethral sphincter activity. Cumulative dose of 8-OH-DPAT (0.003–1 mg/kg, i.v.), a selective 5-HT1A agonist, was administered during filling cystometry study. 8-OH-DPAT improved the voiding efficacy through increasing micturition volume and urethral sphincter activity as well as decreasing bladder capacity, residual volume, and peak bladder pressure in diabetic rats (Gu et al. 2012). In a subsequent study by same study group, the effect of (2,5-dimethoxy-4-idophenyl)-2-aminopropane hydrochloride (DOI), 5-HT)2A/2C receptor agonist was evaluated in similar experimental setting. Cumulative administration of (0.01–0.3 mg/kg, i.v.) improved voiding efficacy by increasing micturition volume and high-frequency oscillation activity and by decreasing bladder capacity, peak bladder pressure, residual volume, and contraction duration in diabetic rats (Tu et al. 2015). Targeting 5-HT receptors seem reasonable in the treatment of LUTD in diabetes.

### Adenosine receptors

A beneficial effect of the adenosine receptor antagonist caffeine on voiding dysfunction in diabetes was shown by a research group from China with several studies. Caffeine (10 mg/kg/day, p.o.) and coffee (caffeine dose 286 mg/kg/day, p.o.) treatments were started at the second day of STZ-induced diabetes and performed for 7 weeks in male rats. These preventive approaches decreased bladder weight, treshold volume for micturition, residual volume, and bladder capacity compared to diabetic rats. Impaired neurogenic and acetylcholine-mediated contraction of bladder strips due to diabetes were subtantially rectified in treated rats. Increased cAMP levels in bladder with both coffee and caffeine are another finding that was ascribed to improved bladder function (Yi et al. 2006). The effect of caffeine treatment in STZ-induced diabetic bladder was evaluated at different doses (5 mg/kg/day and 10 mg/kg/day, p.o.) for 8 weeks in female rats. Caffeine treatment markedly reduced bladder weight, bladder capacity, voiding time, peak voiding pressure, and residual urine volume compared to diabetic rats, and these effects were comparable at both doses. Peak voiding pressure and voiding efficacy were significantly improved in rats treated with both doses of caffeine, yet the effect was greater at 10 mg/kg/day caffeine (Liu et al. 2019). In a further study, female STZ-diabetic rats were treated with caffeine (10 mg/kg/day) for 16 weeks (Xue et al. 2021). Caffeine treatment was found to improve voiding dysfunction by increasing expression of NGF, brain-derived neurotrophic factor, and calcitonin gene-related peptide in bladder tissue and by reducing apoptotic cells in dorsal root ganglion. Reduced antiapoptotic protein Bcl-2 level in diabetic rats was markedly increased with caffeine treatment, and caffeine decreased the level of pro-apoptotic proteins such as Bax, cleaved caspase-3, and cleaved caspase-9 in dorsal root ganglion (Xue et al. 2021).

### Soluble guanylyl cyclase

Soluble guanylyl cyclase (sGC) is an enzyme which is activated by nitric oxide (NO). Activation of sGC increases cellular cGMP levels and mediates tissue specific responses. cGMP mediates relaxation in bladder and other smooth muscle preparations. Thus, the NO-sGC-cGMP pathway may be important in the treatment of voiding and storage disorders (Rahnama'i et al. 2013). HFD-fed obese mice had increased voiding frequencies, nonvoiding bladder contractions, and receptor-dependent (induced by carbachol) and receptor-independent (induced by KCl and CaCl_2_) bladder contractile responses (Leiria et al. 2014). These results were accompanied by a decreased expression of sGC α1 and β1 subunits and cGMP levels in bladder tissue. Oral administration of 1 mg/kg BAY 60–2770 (an sGC activator) to obese mice for 2 weeks fully reversed the above changes in the bladder (Leiria et al. 2014). In another study, the mRNA expression of PDE5 was increased, while cGMP levels were lower in the urethra in STZ-induced 8-week T1DM rats than those in the control group (Gotoh et al. 2021). Urethral pressure reduction and high-frequency oscillation amplitude during voiding were decreased in T1DM rats, indicating impaired urethral relaxation. sGC activation (BAY 60–2770, 1 mg/kg/day for 2 weeks, oral) reversed the changes of PDE5 expression and cGMP levels and improved cystometric (opening pressure, closing pressure and % voiding efficiency) and urethra perfusion parameters (urethral pressure at the beginning of relaxation, urethral pressure reduction and high frequency oscillation amplitude) in diabetic animals (Gotoh et al. 2021). These studies demonstrated that activation of sGC is an effective option for the treatment LUTD in DM. However, more studies are needed to clarify their effect and efficacy in diabetes related LUT dysfunction.

### Myoinositol

Myoinositol, the most common isomeric form of inositol, is naturally found in plant and animal cells and also is produced from glucose in human cells. Myoinositol has various beneficial effects in diabetes including improvement of insulin sensitivity by showing an insulin-like effect (Croze and Soulage 2013). In addition to its insulin sensitizing effect, myoinositol has a beneficial effect on hyperlipidemia which is a common feature of type 2 diabetes (Antony et al. 2017). Myoinositol was shown to increase insulin sensitivity through upregulation of peroxisome proliferator-activated receptor γ and the glucose transporter GLUT4 in adipose tissue of HFD + STZ-induced T2DM rats (Antony et al. 2017). On the other hand, accumulation of sorbitol in cells due to hyperglycemia subsequently leads to decreased myoinositol levels in diabetes. There are limited studies related to the effect of myoinositol on LUT dysfunction due to diabetes. STZ diabetic rats received 1 g/kg/day of myoinositol via drinking water for 8 weeks beginning 3 days after STZ injection (Gousse et al. 1991). Myoinositol treatment did not restore blood glucose levels, reduced prostate size, or reduced prostate β-adrenoceptor density compared to diabetic animals. A study of similar design investigated the effect of myoinositol on muscarinic receptors in the prostate. Diabetes resulted in reduced prostate size and downregulation in prostate M_3_ receptor level, and myoinositol treatment did not improve either parameter (Latifpour et al. 1991). In a subsequent study by the same group, myoinositol showed no effect on increased bladder size and increased bladder muscarinic receptor levels in STZ induced diabetic rats (Latifpour et al. 1992).

### Daidzein

Daidzein is an isoflavone mostly found in soybean, legumes, and soy-based products. It also known as a phytoestrogen due to its similar chemical structure with mammalian estrogen (Alshehri et al. 2021). Estrogen receptors were shown to mediate cytoprotective effects of daidzein in vitro (Xu et al. 2009; Robb and Stuart 2014). The effect of daidzein in diabetes and in diabetes-related complications gained attention in recent years. Daidzein exerted beneficial effects on glucose and lipid homeostasis in T2DM mice models (Park et al. 2006; Cheong et al. 2014). Daidzein prevented onset of diabetes in nonobese diabetic (NOD) mice (Choi et al. 2008) and promoted significant decrease in postprandial glucose levels in STZ-induced diabetic mice (Park and Ju 2013). Daidzein induced PPARγ activation which leads enhanced insulin sensitivity and glucose uptake in murine 3T3-L1 adipocyte cells (Cho et al. 2010). Anti-inflamatory and/or antioxidant effects of daidzein were shown in diabetes’ several related complications such as endothelial dysfunction, neuropatic pain, retinopathy, and cardiomyopathy (Roghani et al. 2013; Jia et al. 2020; Laddha and Kulkarni 2021a, 2021b). Besides, daidzein showed antitumorigenic activity against bladder carcinoma (Su et al. 2000; He et al. 2016). Urinary bladders from Wistar male rats subjected to in vitro anoxia-glucopenia and subsequent reperfusion. Incubation with daidzein, 60-min before anoxia-glucopenia, during anoxia-glucopenia, and first 30-min of reperfusion, resulted in reduced electrical field stimulation-induced contraction concentration dependently. In the same study, rats were received daidzein treatment (2 or 20 mg/kg/day, s.c.) for 1 week. At the end of treatment, bladder tissue exposed to anoxia-glucopenia and reperfusion. Electrical field stimulation-induced contraction responses in low-dose daidzein-treated rats were found significantly higher. Moreover, malondialdehyde generation in treated rats were found lower. Combining the direct relaxant effect of daidzein against electrical field stimulation-induced contraction in detrusor strips, with neuroprotective and antioxidant effect of the compound, may be potential treatment strategy in LUT dysfunction (Valeri et al. 2012). Daidzein was shown to be a promising treatment agent against the complications of diabetes in several studies. However, little is known on diabetic LUT function. Considering its beneficial effects in diabetes and on bladder, this venue is worth to be elucidated. Recent work indicates that treatment with daidzein in the rat STZ model of diabetes prevented the loss of antioxidant enzyme activity and attenuated the elevated expression of NADP oxidase and Rac1 in the bladder; concomitantly, it improved bladder capacity, voiding efficiency, and residual urine in cystometric studies and improved mucosal lymphocyte infiltration and multifocal hyperplasia of the transitional epithelium (Laddha and Kulkarni 2022).

### Nonpharmacological interventions

Some patients may not benefit adequately from established pharmacological treatment approaches used in diabetes-related LUTS. Therefore, nonpharmacological interventions could be a choice of therapy in these patients.

#### Stem cell therapy

Stem cell-based therapy approaches have gained popularity in recent years to treat a variety of diseases including untreatable disorders (Zakrzewski et al. 2019). Different types of stem cells were evaluated in diabetic bladder dysfunction. Adipose-derived stem cells was injected either into the tail vein (DM + T group) or into the detrusor (DM + B group), 3-month after HFD + low-dose STZ diabetes induction in female rats. One month after treatment, bladder function was evaluated. Reduced micturition interval and urine volume per void were improved voiding function which was increased by 40% in DM + T group and by 60% in DM + B group compared to diabetic group. Adipose-derived stem cells promoted angiogenesis and inhibited apoptosis in vivo and in vitro, which were postulated to be the underlying mechanisms of its beneficial effect on diabetic bladder function (Zhang et al. 2012). Human embryonic stem cell-derived multipotent mesenchymal stem cells (M-MSCs) at three different doses (0.25, 0.5, and 1 × 10^6^ cells) were transplated to STZ-induced diabetic female rats 3 weeks after diabetes induction. Bladder function was assessed 1, 2, and 4 weeks after M-MSCs transplantation. Bladder underactivity that worsened time dependently was observed in diabetic animals. M-MSCs improved contractile function and voiding efficiency of bladder. Myogenic activity of bladder was restored through integration of M-MSCs into pericytes. M-MSC treatment was rectified increased fibrosis, apoptosis and mast cell infiltration due to diabetes as well (Shin et al. 2020). In another study, STZ-diabetic male rats were administered integrin-linked kinase gene-modified bone marrow-derived stem cells intravenously at 2-week after diabetes induction. Four weeks after, abnormalities in bladder function was improved with gene-modified stem cell treatment. A beneficial effect of integrin-linked kinase gene-modified stem cells was attributed to reduced apoptosis and increased proliferation, migration and angiogenesis via distinct molecular pathways (Huang et al. 2020). Besides the well-known types of MSCs, the effect of a new type of MSCs, neonatal bladder-derived mesenchymal stem cells (nMSC-B), was evaluated in diabetic bladder. nMSC-B was administered either i.p. or directly to the bladder wall to 12-week STZ diabetic male rats and 4 weeks after application bladder function was evaluated. nMSC-B were found to be effective to restore bladder function (Boga et al. 2020). Beneficial effects of different stem cell therapies in diabetic bladder dysfunction ascribed to its paracrine effect (Zhang et al. 2012; Huang et al. 2020; Shin et al. 2020). Stem cell therapy stands as a promising approach in diabetic bladder treatment that needs to be supported by further clinical tirals.

#### Low-intensity extracorporeal shockwave therapy (Li-ESWT)

Li-ESWT is a noninvasive method which has been shown to be a promising treatment approach in several diseases including urological disorders (Lin et al. 2021). Recently, the effect of Li-ESWT in the treatment of bladder dysfunction was invastigated in different studies. Li-ESWT was applied once weekly for 4 weeks to STZ-diabetic female rats 4-week after diabetes induction. Cystometry analysis was revealed increased postvoid residual volume, voided volume, micturition interval and decreased maximal detrusor pressure due to diabetes, and Li-ESWT ameliorated postvoid residual volume and maximal detrusor pressure to some extent. Contractile function of bladder was abolished in response to electrical-field and muscarinic receptor-mediated stimulation in diabetic animals, Li-ESWT restored the contractile activity. Additionally, morphological and neurological abnormalities in bladder were abundantly improved by Li-ESWT (Wang et al. 2018). In another study, Li-ESWT was given to HFD (4-week) + low-dose STZ diabetic female rats 1-week after STZ injection, twice a week for 4-week. Li-ESWT was markedly improved micturition interval and maximal detrusor pressure which were reduced due to diabetes. Besides improving voiding function, Li-ESWT was restored nerve expression and was activated angiogenesis in bladder tissue (Lee et al. 2021). Beneficial effect of Li-ESWT in diabetic bladder was attributed to reduced gene expression of transient receptor potential vanilloid 1, interleukin-1β, the muscarinic receptors M2 which are related to mechanosensation, ischemia/inflammation and bladder contraction respectively. Li-ESWT did not affect other muscarinic receptor subtype M1 and M3 expression (Dimitriadis et al. 2021). Li-ESWT may be a promising approach to restore bladder function in diabetic patients.

#### Neuoromodulation

As previously stated, neuropathy is one of the underlying causes of diabetes related bladder dysfunction. Neuromodulation was shown to restore voiding function in several studies (Chen et al. 2013; Hsieh et al. 2016; Han et al. 2021). Voiding efficacy in control animals was found about 75% while it was measured as 30.02 ± 7.89% after 8 weeks of diabetes and 19.98 ± 6.03% after 18 weeks of diabetes in STZ-induced T1DM male rats (Chen et al. 2013). These data shows that time-dependent reduction of bladder function due to diabetes. Besides reduced voiding efficiency, diabetic animals showed increased volume treshold, contraction amplitude, residual volume, bursting period and had decreased intercontraction interval, voided volume. Acute electrical stimulation of pudendal sensory nerve at low intensity (0.025–0.05 mA) resulted in an increase in voiding efficacy only in 8-week diabetic animals. When, intensity of electrical stimulation was elevated to 0.1–0.3 mA, voiding fuction was restored in both 8-week and 18-week diabetic animals by reduced residual volume and increased intercontraction interval, contraction duration and bursting period. Following 0.1–0.3 mA electrical stimulation voiding efficiency was markedly increased to 40–50% but was not reach to control levels (Chen et al. 2013). In another study, 4 days after induction of diabetes with STZ injection, female rats were subjected to chronic electrical stimulation (3 or 6 weeks) (10 µA) of pudendal sensory nerves . Voiding efficiency in control rats was about 64%, which decreased to 45% and 17% in 3 weeks and 6 weeks of diabetes, respectively. Chronic electrical stimulation therapy resulted in better voiding function, which was increased from 45 to 57% with 3 weeks and from 17 to 51% with 6-week of application. Electrical stimulation improved abnormalities in bladder function parameters such as contraction duration, residual volume, bursting period independent of its duration. Axonal desity of pudendal nerve was markedly decreased in 6-week diabetic animals which was reversed by chronic electirical stimulation (Hsieh et al. 2016). Electroacupuncture treatment for 8 weeks was eveluated in a T2DM model induced by combination of HFD (4-week) + STZ injection. At the end of 12 weeks, diabetic animals presented with bladder hypertrophy, increased residual volume and volume threshold for micturition. All of the abnormalities were improved by electroacupuncture therapy and its beneficial effect was ascribed to regulation of myosin light chain and myosin light chain kinase phosphorylation. Moreover, electroacupuncture was reduced infiltration of inflammatory cells in bladder (Han et al. 2021). These studies suggests that electrical stimulation of bladder may prevent or improve bladder dysfunction in diabetic individuals by facilitating bladder contraction.

## Conclusions

The prevalence of diabetes and hence its undesirable effects increases continuously and affects millions of people worldwide. Diabetes-related macrovascular and microvascular diseases and treatment approaches are widely studied due to their high morbidity and mortality rate. Nevertheless, LUTD related to diabetes is a very common complication that considerably impairs quality of life. Thus, efficient treatment approaches are needed for diabetic people who suffers from LUTD. Considering the mainstay LUTD medications in diabetes, α_1_-adrenoceptor antagonists have comparable effect in clinical studies in diabetic and nondiabetic patients, whereas preclinical studies show inconsistent findings. Based on few studies, 5α-reductase inhibitors may worsen the diabetes-associated LUTD. In preclinical studies, the PDE5 inhibitor tadalafil was shown to have beneficial effect against LUTD in diabetes. Muscarinic receptor antagonists were found to be a little less effective in the treatment of OAB with concomitant diabetes in preclinical and clinical studies, however further assessment is needed for conclusion. The effect of β_3_-adrenoceptor agonists on LUT function in diabetes setting need to be studied.

There are few clinical studies investigating the possible effects of antidiabetic treatment agents on LUT dysfunction in T1DM and to the best our knowledge, there are no studies in T2DM. Moreover, most of the preclinical studies have been conducted on animal models of T1DM. Considering the high incidence of T2DM over T1DM, findings from preclinical studies remain insufficient for clinical translation. Insulin was shown mostly to prevent diabetes-associated LUT dysfunction as well as can reverse the symptoms. Metformin showed some beneficial effects on LUTD in normoglycemic animals. Effect of other T2DM medications (SGLT2 inhibitors, dipeptidyl peptidase-4 inhibitors, GLP-1 agonists) on LUTD in diabetes remains to be studied. Based on our search, there might be possible novel pathways involved in the development of LUTD in diabetes and promising candidate compounds in the treatment of diabetes-associated LUT dysfunction. In preclinical studies involvement of oxidative stress, inflammation, GTPase Rac 1, NGF, angiotensin II, 5-HT and adenosine receptors and sGC in LUT function have been demonstrated in normoglycemic and/or hyperglycemic conditions. Hence, compounds targeting these pathways may be beneficial in the treatment of diabetic LUTD. However, data on diabetes related LUTD either are few and inconsistent or do not exist. Thus, more studies are needed with these compounds. Other than pharmacological treatments, nonpharmacological interventions such as stem cell therapy, Li-ESWT and neuoromodulation were found to be effective in diabetic bladder treatment in preclinical studies. These approaches may be a promising alternative for those patients who can not benefit from standart trerapy regimen.

Our selective literature search found an insufficient number of preclinical and clinical studies on either LUTD medications in diabetes or antidiabetic treatments on LUTD to derive robust conclusion. Inconsistent data or low clinical relevance due to study structure make interpretation of the data difficult. There are points to be elucidated in this area by further research. Hence, properly conducted preclinical and clinical studies are required to meet the need in this field.

## Abbreviations

BPH: Benign prostatic hyperplasia
DM: Diabetes mellitus
GLP-1: Glucagon-like peptide 1
HFD: High fat diet
IPSS: International Prostate Symptom Score
Li-ESWT: Low-intensity extracorporeal shockwave therapy
LUT: Lower urinary tract
LUTD: Lower urinary tract dysfunction
LUTS: Lower urinary tract symptoms
M-MSC: Multipotent mesenchymal stem cells
nMSC-B: neonatal bladder-derived mesenchymal stem cells
NGF: nerve growth factor
NO: Nitric oxide
OAB: Overactive bladder syndrome
PDE5: Phosphodiesterase type 5
ROS: Reactive oxygen species
SGLT2: Sodium-glucose transporter 2
sGC: Soluble guanylyl cyclase
STZ: Streptozotocin
T1DM: Type 1 diabetes mellitus
T2DM: Type 2 diabetes mellitus
TNF-α: Tumor necrosis factor-α
UAB: Underactive bladder syndrome
